# Differentiations in Visibility-Male Advantages and Female Disadvantages in Gender-Segregated Programmes

**DOI:** 10.3389/fsoc.2020.563204

**Published:** 2020-10-07

**Authors:** Mia Heikkilä, Anna Isaksson, Frida Stranne

**Affiliations:** ^1^Faculty of Education and Welfare Studies, Ȧbo Akademi University, Turku, Finland; ^2^School of Health and Welfare, Halmstad University, Halmstad, Sweden; ^3^School of Education, Humanities and Social Sciences, Halmstad University, Halmstad, Sweden

**Keywords:** gender segregation, gender minority, higher education, experiences, visibility, sense of belonging, negotiating otherness

## Abstract

This article stresses the importance of understanding that women and men in gender-segregated programmes experience their gender minority positions very differently. It stems from an interest in the kind of interventions that academia should address in order to reduce gender segregation and provide women and men with the same educational opportunities and personal development. In relation to the obvious and continuing gender differences along a horizontal dimension, previous research seems to have had a limited impact in breaking gender stereotypes and promoting women and men to more atypical fields. The empirical data consists of 25 semi-structured, individual interviews from underrepresented students' gender-related experiences/thoughts about their programmes. By using the concepts of “visibility,” “sense of belonging,” and “negotiating otherness” to analyze how negotiation and belonging are part of students' everyday university lives this study's most important contributions are its findings regarding the differentiations in visibility. A continuum of visibility experiences is explored, from men who receive positive attention to women who are being considered as less knowledgeable. Our visibility scale indicates, as does previous research, that there are differences between how female and male students become visible, but the differences can also appear within both groups of students. This knowledge is crucial when designing interventions so as to provide positive study environments for both women and men. Also—in a broader perspective—it is important in order to recruit and ensure that gender minority students remain in the programs.

## Introduction

The main objective of Sweden's gender equality policy is that women and men shall have the same possibility to shape the society and their own lives. Further, the government has six sub-goals to achieve gender equality. One is “equal education:” The government states that women and men, girls and boys should have the same opportunities and conditions with regard to education, study options, and personal development (www.regeringen.se). But gender differences persist along a horizontal dimension. Decades of research and evaluations have documented that despite policies and efforts to lessen gender segregation in Swedish higher education, women, and men continue to enroll in different study programmes and subjects, based on their gender. Women still dominate in health care, elementary education and domestic spheres (HEED), while men dominate in science, technology, engineering and mathematics (STEM) (UKÄ (Swedish Higher Education Atuhority), 2016).

This study stems from an interest in the kind of interventions that academia should address in order to reduce gender segregation and to meet the equal education sub-goal that is given by the government, as well as provide women and men with the same educational opportunities and personal development. Given long-existing patterns, higher education institutions have not been able to break the history of gender stereotyping and the fact is that women and men continue to choose subjects according to traditional gender roles. Thus, policy makers, teachers and parents must tackle gender stereotypes early on. However, universities can affect how students experience and perceive their study and learning environment from a gender equality perspective; also, they can encourage students to choose programmes other than those that typically have been selected due to gender stereotypes. In fact, Swedish universities are, according to the Swedish Discrimination Act, required to take active measures to prevent discrimination and ensure that the study environment (physical, psychological, and social circumstances) is safe, equal, inclusive, and facilitates the studies.

Previous research (e.g., Steele et al., 2002; Honghong et al., 2011; Fouad et al., 2016; Heikkilä, 2016) has focused on how women and men in gender segregated programmes experience their study environment. However, in relation to the obvious and continuing gender differences along a horizontal dimension, this research seems to have had a limited impact in breaking gender stereotypes and promoting women and men to more atypical fields. Thus, this article will study how gender minority groups in highly gender-segregated programmes experience their study environment; and, the aim is to suggest the measures and interventions higher education institutions should address to provide equal study and learning environments to lessen gender segregation and create change. The research questions that are explored are the following:

How do women and men in gender-segregated, higher-education programmes experience their studies and study environment?

The study examines underrepresented students' gender-related experiences in four vocational programmes at a university in Sweden: nursing programme (12% of the students were men), pre-school teacher programme (5% of the students were men), construction engineering programme (29% of the students were women), and computer science and engineering programme (15% of the students were women). When we write about “gender minority groups,” we refer to numeric minority, i.e., women studying in male-dominated STEM-fields and men studying in female-dominated HEED-fields.

## Previous Research

### Educational Experiences for Gender Minority Students

Ecklund et al. (2012) argue that gender represents a more important reason for choosing a field of study than the area itself. They claim that interest is not the primary reason for one's choice, but rather is related to gender norms. Snyder and Green (2008) show that men working in nursing are drawn to areas that are considered more masculine, such as emergency care and anesthetics. Tellhed et al. (2017) found that beliefs about one's capabilities and social expectations (where people fit in socially) affect gender differences in the various sub-specialties.

Gender differences in STEM fields relate strongly to women's low self-confidence in these areas and to a lesser degree the question of social belonging. For male students, the issue of social belonging partly explained their reduced interest in HEED studies, while self-confidence did not factor in as a reason for not choosing to major in these fields (Tellhed et al., 2017). The men in HEED-professions were given more promotional advantages, though also expected to do traditionally masculine duties such as heavy lifting (Williams, 1992).

Other studies demonstrate how women's educational experiences are affected by gender: Several have noted the problems and obstacles they encounter in engineering programmes (e.g., Powell et al., 2009; Morganson et al., 2010; Singh et al., 2013; Fouad et al., 2016). Further, it has been argued that women doubt their abilities to a greater extent than men in male-dominated educational fields, although no differences in performance have been demonstrated. The limited research on men in gender minority fields shows they do not doubt their capacities to the same extent (Cheryan and Plaut, 2010).

Cech et al. (2011) examine how women in male-dominated subjects tend to lack professional role confidence because they lack external encouragement. Professional confidence can be understood as an individual's confidence in his/her ability to successfully complete tasks, and develop a positive identity in a profession. Male students usually get such encouragement readily, but women feel they must search for it. Moreover, the lack of confidence reduces their likelihood of staying within these professions. Thus, women studying engineering need to be included on equal terms and also encouraged in the same ways as the male students.

A study by Steele et al. (2002) found that women who study in male-dominated fields in the U.S. experience more difficulties (due to their gender) than men. Women experienced gender-threats and discrimination and considered leaving their programmes. Conversely, men who were in the minority said they did not experience threats to the extent that women did.

Despite years of research aimed at understanding why women are underrepresented in STEM fields, fewer efforts have been made to understand why men are underrepresented in HEED fields (Block, 2015). There is also a lack of research on and attention to men's experiences in gender minority positions in HEED programs—with some notable exceptions. For example, Buthelezi et al. (2015) describe the learning experiences of male nursing students. Referring to previous studies (Levett-Jones et al., 2008; Mabuda et al., 2008; Pitkäjärvi et al., 2012), they say there seem to be fewer differences between male and female experiences as nursing students; however, Buthelezi et al. (2015) demonstrate how male nursing students experience more challenges than the females in clinical settings, which significantly affect their self-esteem. The authors suggest that male students should be provided with more support during their training, to help them build confidence.

Keogh and O'Lynn (2007) explore the consequences of non-supportive learning environments. In their study, they describe how faculty and staff nurses tend to be negative toward male students, which can cause them stress. Honghong et al. (2011) describe how male nursing students experience loneliness and psychological stress. This is also noted by Stott (2007) who found that male nursing students felt isolated. In addition, Kleinman (2004), Li and Ren (2007), and Stott (2007) found that male students, as a minority in nursing programmes, avoid interacting with their female classmates, fearing they will be regarded as feminine.

Heikkilä (2016) shows similar patterns regarding males in pre-school teacher programmes. She found that male students do not want to be identified as *male* preschool teachers, but as preschool teachers, without the prefix “male.” These concerns did not affect their self-confidence, since they were warmly welcomed in the schools. However, male students at pre-school programmes tend to drop out to a greater extent than women (UKÄ (Swedish Higher Education Atuhority), 2016). A number of studies have concluded that the risk of being accused of child sexual abuse causes anxiety and stress among male students (Nordberg, 2005; Heikkilä, 2016).

To summarize, students who study subjects and are enrolled in programmes that are atypical for their gender encounter various obstacles such as isolation and discrimination and suffer from stress. In previous research, it has been suggested that these obstacles could be reduced by supporting the gender minority students through social activities and programmes and/or to promote self-esteem. However, few studies compare different experiences and programmes in the same study which means that this study has important contributions to make.

### Theoretical Framework

Gherardi and Poggio (2001) speak about organizations that are “doing gender” in a way that is applied in this study. This is a way to understand the role of gender in organizations such as universities, as a continuous process that affects the participants. Gherardi and Poggio (2001, p. 248) describe it in the following way:

“By saying that also organizations ‘do gender’ we mean that organizational cultures contain specific rules, values, meanings expressed in social situations in which gender-positioning processes are realized as interpersonal relations in a public process whereby gender meanings are progressively and dynamically achieved, transformed, and institutionalized. It should be emphasized that gender is not located solely at the level of interactional and institutional behavior (the gender we do); it also lies at the level of symbolic structures (the gender we think).”

This approach can be linked with a perspective of how learning is a central activity for universities and how “doing learning” constantly needs to be understood in relation to “doing gender,” or perhaps more widely “doing identity.” Identity work in these contexts can be seen as “a set of active processes (such as forming, strengthening and revising) which serves to construct a sense of identity” (Beech, 2008, p. 51), which consists of activities and negotiations that are constantly carried out in social contexts.

Nentwich (2006) suggest a typology of doing gender, where it is divided into five aspects of empirical “evidence.” In this study, Nentwhich and Kelan's concepts are not applied but they form a clear focus of how to empirically study “doing gender.” They consider the concepts of negotiation and belonging, which, to Nentwhich and Kelan, could be a mix of doing gender through structures and identity work. The concept “sense of belonging” (c.f. Hattie, 2009) is a way of capturing the aspects of learning in the empirical data together with “negotiating otherness” (Sumsion, 2000), which can be connected to understanding how gender is being done in a minority position.

This study was conducted at one university where the main aim was for the students to develop knowledge in different subject areas to enable them to practice a specific profession in the future. Learning is the formal focus of why students are at the university, but in order for it to occur, the environment needs to be permissive and inclusive, where students are given a sense of belonging (Hattie, 2009), which can contribute to a basic security that allows them to try and test new and old concepts, reject incorrect information and acquire new information. These are central components of learning processes (Vygotskij, 1978; Säljö, 2000) that need to be present in supportive educational environments, which include lively communication and interactions—which are crucial in “doing learning” contexts (Vygotskij, 1978).

In the process of “doing learning,” “doing gender,” and “doing identity” power structures are established through the rituals, rules, negotiations, and positionings that occur (Wernersson, 2009; Francis et al., 2012). These can be either constructive or destructive (Selander, 2017). Indeed, the “doings” (the processes) where destructive power is distributed can negatively affect individuals and recreate gendered organizations that exclude and diminish individuals and groups.

### Analytical Concepts

The analytical concept adopted in this study enables an understanding of how the learning context is formed of gender relations. Together with processes of doing gender, identity work in terms of negotiating social positions (which result in a sense of belonging) *visibility* can be a fruitful concept to use to understand how these processes are being materialized into social university life. Being visible to one self and others is also a way to create a feeling of belonging to a group or to a social context.

The concept of belonging is closely linked to the overall educational reasoning above. To belong in a social context can be understood as closely related to being and becoming visible and making oneself visible. What can be added is how individuals of one gender can feel a sense of belonging, although they are a distinct minority, depending on how the majority group receives and includes them in terms of visibility. If there is a strong normative formation of the majority group, it is unlikely that minority individuals feel they belong to it. “Negotiating otherness” (Sumsion, 2000) can be understood as realizing you are in a gender minority position, reminded of this by oneself and others and thus negotiate how to make that difference visible. It is important that both the “sense of belonging” and “otherness” are ongoing negotiations: They are not stable or states that can be determined by others. For example, being a male in a female-dominated programme means negotiating your identity, rights, and obligations in relation to others.

Combining these concepts makes it possible to discuss elements of gender minority positions in the university context: i.e., it raises the question about what it is like to not to fit in properly, not belonging to the social norm, and how individuals handle the social structures in which they participate.

### Design of Study, Methods of Data Collection and Data Analysis

The purpose of this study is to capture underrepresented students' (male and female) gender-related meanings, experiences, and thoughts about the content and structure of their programmes, their everyday life as university students and thoughts about being in a gender minority position. Thus, we apply a qualitative approach since it will be able to capture experiences, meanings, and thoughts (c.f. Brinkmann and Kvale, 2018; Cohen et al., 2018).

The empirical data consists of 25 semi-structured, individual interviews from underrepresented students' experiences and thoughts about their studies and study environment. Each interview followed a specific questionnaire (Appendix 1). The interview guide contained themes and questions all of which were asked to each student. However, the interviews were flexible as questions were adapted and changed depending on the students' answers.

The four vocational university programmes included are (a) nursing programme (b) preschool teacher programme, (c) construction engineering programme, and (d) computer science and engineering programme. Each has a clear gender majority but the pre-school teacher and computer science and engineering programmes are more gender-segregated than the constructions engineering and nursing programmes. Students, who were selected with information from programme managers, were informed about the study from one of the project members by email or telephone.

For ethical reasons, the exact number of interviews from each programme will not be presented since they involve very few gender minority students, who could thus be easily identified (c.f. Berg and Lune, 2012). Women in the civil engineering and computer science programmes were interviewed; men in the nursing and pre-school teacher programmes were interviewed. All together 13 male students and 12 female students were interviewed. The women were 21–28 years of age. The average age among the female students was 24 years. Many of the female students came to the university directly from upper secondary school and had not worked or taken time off from their studies. The men were 21–42 years. The average age of the male students was 30 years. All interviews were conducted by a research team member. Each interview took between 25 and 60 min, took place at the university in a meeting room, and was transcribed; and, the team used the transcripts to make the analysis—although the audio files were available, if needed.

### Analysis Process

The analysis, whose focus was to understand the gendered everyday experiences of those interviewed at the university, involved several steps of coding. It was inspired by the Constructivist Grounded Theory (CGT). In CGT, knowledge is considered socially constructed and developed through joint interpretation (c.f. Charmaz, 2008, 2014).

Our empirical data was analyzed through initial coding, focused coding, and coding of subcategories. Through the analysis, comparisons were made between data, memo writing, and theoretical framework/concepts (c.f. Charmaz, 2014). This could be considered to be a deviation from one of the main principles of grounded theory. Traditionally, grounded theory sets out to construct theory from empirical data. However, in our case, we applied our theoretical framework and analytical concepts while categorizing our empirical data. This demonstrates how grounded theory can be adapted in various ways. Bryant and Charmaz (2007) argue that grounded theory strategies have almost become routine practices in qualitative inquiry. Qualitative researchers adopt aspects and coding strategies from grounded theory for coding and synthesizing data and developing themes. As demonstrated above we have been using some of the elements from CTG, i.e., coding techniques for our data analysis.

## Results

### Always Gender, Always Visible

Visibility seems to be a crucial factor for most of the gender minority students, something they had to routinely consider. Based on the model developed by Morgan and Davis-Delano (2016) and Chatfield (2018), Table 1 summarizes our results.

**Table 1:** “Visibility” differentiations.

**Subthemes of visibility**	**Description of theme**	**“Visibility scale”**
Experiencing appreciation	Students' examples of how they were appreciated for choosing a field of study that is atypical for their gender	Only men were in this category, most of whom were nursing students, but some were also pre-school students
Being neutral to the visibility	Students' examples of how gender does not affect their studies	Only men were in this category; most were pre-school students, but some were nursing students.
Experiencing negative attention	Students' examples of negative attention related to gender because of their professional choice	Mostly women were in this category; most were constructing engineering students, but some were computer science and engineering students
		Some male pre-school students note negative attention but not in the study environment itself. They are afraid of being accused as pedophiles when they start working.
Being considered less knowledgeable	Students' examples of how their knowledge is questioned by other students due to their gender	Only women were in this category, all of whom were in computer science and engineering

The concept of visibility is divided into four subthemes, which illustrate its differences and consequences. These are labeled *experiencing appreciation* when visible, being neutral to the *visibility*, experiencing *negative attention*, and being *considered less knowledgeable*. These four categories create a polarity where women's experiences are mostly negative, and men's experiences are positive. In addition to these categories, a pattern emerges in which students in a highly gender-segregated programme, where the gender minority group is very small, experience greater visibility than those in a programme with less gender segregation. Among the categories there are ongoing negotiations and the categories can be seen as social signs of negotiation. Some students change positions between categories, which can be understood as a way of continually negotiating *otherness* and thereby also negotiating how one's identity is “allowed to” be displayed.

### Experiencing Appreciation

Being visible includes getting attention and often appreciation, at least for some. This appear as a clear pattern in the interviews with male students. The male students on the pre-school teachers programme and nursing programme indicate that they are getting a very positive response during their internship because they are men. Here is how one male student expresses it;

“Many older people express ‘wow, a man is coming now, a guy is coming to my room now and taking care of me,’ ‘oh come here’ like this. They behave in this way.”

Male nursing student

There are several similar statements in the empirical data, for example;

“So, everyone is really positive that I come to an internship as a male preschool teacher student. Both the parents and the children appreciate it very much.”

Male pre-school teacher student

However, there are male students who express a hope of also being appreciated as just preschool teachers, and not just as *male* preschool teachers.

Visibility can also be an expectation that one is a hero or pioneer, which is a form of appreciation.

“They always look up to you when you come out. So that's an advantage, I think.”

Male nursing student

In this example the student link visibility to advantage, and this can be interpreted as something he wants to keep.

Women that were interviewed do not express that they encounter this kind of appreciation. They do not experience appreciation of the professional choice they made, although in the societal discourse it is often argued that more women are needed, for example, in the engineering profession. The female students are capable to reflect upon the fact that more women in the engineering industry are needed, but they do not describe this in the same way as the male students' experience of their professional choice.

### Being Neutral to the Visibility

On the other hand, in some cases, gender does not seem to matter in the male students' everyday practice and study environment. In the interviews we find students who “don't care” about being visible or who don't mention aspects that can be understood as related to visibility. As mentioned above, the preschool students do not want to be considered as *male* preschool teachers. Otherwise, gender does not seem to be something causing negative and/or positive experiences. The preschool students express their experiences of visibility rather neutral. Gender is always present. It is integrated into the subject content, but not an “issue” in the classroom and study environment.

“Of course, it is obvious that you are in a gender minority. But I think it is ok being a male student and a future preschool teacher.”

Male pre-school student

The male nursing students also describe how gender is present most of the time but not really an issue. The visibility is neither negative nor positive. However, one of the nursing students expresses that even if he can not report any negative experiences, he would feel more comfortable if the nursing programme would be less gender-segregated;

“It would be more secure for me to have more guys around and not so many women around me. Even though I communicate better with women than men. But… I cannot really describe the experience, but it would feel better to have more guys.”

Male nursing student

### Experiencing Negative Attention

For other students, namely women in gender minority groups, visibility involves a negative experience. In this group the students do not like being visible; they are worried about what happens if they are too visible and for turning out as not smart despite being visible. It can be understood that, if they are not perceived as smart, they deviate from the expected norms of being women in education. Here are two different female students' voices;

“You do not want to be seen as unknowing because you are expected. yes, it is actually that you are expected to be smart as a woman.”

Female construction engineering student

“Once we had a guest lecturer who was there for some time, it was something about electrical engineering, so he explained and drew up a schedule on the board, and he began to talk a little about his wife, arguing she couldn't easily understand the schedule, so he said something like ‘yes, it is a little harder for women to understand something like this.’ And I just… I said something like just what do you mean there, how can you say that? All the other girls in my class just sat completely silent, they did not seem to care as I did. I got upset, how can you stand there saying that.”

Female construction engineering student

It is only women who describe this kind of anxiety, even though men to a certain extent on the preschool teacher programme also express similar worries. However, the material stresses that women dominate regarding experiencing visibility as something negative and as something that causes concern and anxiety.

This concern also extends beyond the university, and includes an imagined future, continuing to be a visible person, even at a future workplace. One of the students expressed concerns about entering the industry she is studying to work at. She worries about how she will fit into a workplace where the majority are men.

Another student expresses the visibility and the vulnerability like this:

“As I said, it was a bit more difficult to blend in at the beginning. So, you would need to be accepted before you could set limits, and just not say “no, I do not like that” or “that joke is not funny” and so on. But, from the beginning it was a little. a little challenging to do that.”

Female computer science and engineering student

In this example it is primarily male fellow students who make visibility somewhat negative, and this female student adopted the strategy of accepting how the majority behaved in order to be accepted and included. Slowly she started to re-negotiate that position and find a new way of being herself.

### Being Considered Less Knowledgeable

The last theme—considered unknowing—has similarities with experiences of negative attention but also add how visibility is interwoven with vulnerability and harassment. This kind of visibility is expressed by women studying at the most gender segregated program, Master of Science in Computer Science programme.

“Because, we are so few, many guys assume, I think, that girls do not have the same skills in technology, and then you become a fairly easy victim. Or, suppose we don't know as much as they do.”

Female computer science and engineering student

In the interviews women in these group also describe how this require them to perform better. They express how they often try to convince those who ignore them, in order to become accepted and perhaps achieve a sense of belonging in the context. Additionally, many of them, to some extent, take responsibility for their feelings at the same time recognizing that the context and the fellow students who makes them feel unknowing is wrong.

“One example was when I sat and studied alone, and a male student came and started looking at my notebook, just pointing, just saying ‘you’re wrong. And he does not know what assignments I do, he does not know where I am in the course syllabus, he just says I'm wrong. And I knew I was not. And then he came back a while later and just ‘no, you were not wrong.’ So, it feels like some, yes, just want to point out that I cannot be right.”

Female computer science and engineering student

The example tells us there are male students who seem not to be able to accept that women students can have solutions and knowledge of the subject they study. The interviews show that it is usually just male fellow students who question women's knowledge, no other female students or teachers.

However, there are some exceptions when teachers participate in the questioning.

RESPONDENT: …If I've worked on an assignment with a guy, I've been asked whether I've really done parts of the task. I have experienced that several times.

INTERVIEWER: Did you experience that several times? By who then?

RESPONDENT: By the teacher, who was the examining teacher. He's walking around and checking whether you've been working in the laboratory.

INTERVIEWER: In what way, can you express, how did you know, how did you perceive the signals?

RESPONDENT: They have questioned med and if I have done the work, insinuating I have not participated.

INTERVIEWER: And this when you actually have been participating in the same way as the others?

RESPONDENT: Yes, there have even been times when I've done everything.

INTERVIEWER: You have done *everything*?

RESPONDENT: Yes.

INTERVIEWER: Mm. How did that feel? It looks as if you're concerned about it (the respondent starts crying).

RESPONDENT: Yes.

INTERVIEWER: That it had a bad effect on you?

RESPONDENT: Yes.

Female computer science and engineering student

In the expressed situation, the teacher can be said to contribute to that a student can be considered “unknowing.” There are also examples in the empirical data when teachers argue that gender equality will never be achieved and have supported male students in such discussions. The female student is then forced into negotiating her otherness, and her “un-belonging” when the teacher is claiming that she has not been participating. She has to find a strategy for how to convince the teacher that she in fact has participated.

## Discussion

Being in a gender minority position means always being visible in some sense. The “visibility scale” presents how women are more likely to express negative experiences of visibility compared to men who express visibility as something mainly positive and/or report experiences that could be described as neutral. In previous research (e.g., Tellhed et al., 2017) it has been demonstrated how women's lower self-efficacy for STEM careers is an important mediator for them not choosing STEM majors. As for male students, social belongingness expectations explain their lower interest in HEED studies. While women doubt their competence in male-dominated fields (Cech et al., 2011; Tellhed et al., 2017) men do not seem to be concerned about their competence, skills, and future performance in female-dominated areas (Cheryan and Plaut, 2010). Rather, their concerns are about the social aspects of the study environment. The analysis of our empirical data suggests that these concerns are “logical” in relation to the study environment women and men seem to enter when they choose an atypical education program. In accordance with previous research (e.g., Steele et al., 2002; Powell et al., 2009; Morganson et al., 2010; Singh et al., 2013; Fouad et al., 2016) women in STEM educations encounter several obstacles. For example, their competence is questioned, and they experience discrimination, gender-based and stereotypical threats. In this study, these kinds of experiences are reported as well. However, our “visibility scale” suggests that women have different experiences of being visible in the two different education programmes. Female computer science and engineering students experience to a greater extent than construction engineering students: *harassment* and *vulnerability*. They are considered as more unknowing than the construction engineering students as well. At the same time, female students on male-dominated programmes also share a lot of experiences, as the result show. They do not express a total lack or support or positive reception for their career choice, but they have said they are repeatedly questioned and exposed in several different ways.

To use the concept of “sense of belonging,” it can be said that women rarely express a positive sense of belonging in relation to their studies. The most vulnerable students are the students of computer science engineering, where the interviews show a skewing of power distribution in the education practice. The male students, to some extent also male teachers, have the power over how communication positions are created. The women see themselves as being disadvantaged, expressing they must relate to the male students. They do not experience equality between women and men in their everyday practice. This problem is visible, to a certain extent, also amongst women on the construction engineering programme, but to a lesser degree. Women can also be said to have to negotiate their gender more frequently presenting themselves as a kind of neutral person, where gender does not exist, even though it is present all the time.

The differences among the female students could be interpreted as a result of which programme they belong to. The construction engineering programme is less gender-divided than the computer science and engineering programme. This is probably why the women in computer science are in a more vulnerable position. However, differences in numbers do not seem to be able to explain why the male nursing and pre-school students tend to experience visibility in different ways. These education programmes are almost equal when it comes to gender distribution. Our analysis demonstrates that the male students' experiences of being visible are mainly positive. They do not seem to experience the challenges reported in previous research such as lower self-esteem, non-supporting learning environments and isolation (e.g., Kleinman, 2004; Keogh and O'Lynn, 2007; Stott, 2007; Honghong et al., 2011; Heikkilä, 2016). However, the empirical data, summarized in the “visibility scale,” indicates that there are small but significant differences. The nursing students are more represented by the theme *experience appreciation* while more preschool students are found in the theme *being neutral to the visibility*. Male students on programmes where women are in the majority, are often faced with positive comments and inclusion in a future professional community. Male nursing students almost acquire a kind of hero status. According to our interviews, they receive great support and encouragement, which gives confidence. Among male students, there is no doubt about whether they have made the right study choices, compared to a greater hesitation around that decision among some women. Accordingly, they do not question whether they will do a great job, rather the opposite. These students take away a high degree of sense of belonging from the programme. Nevertheless, there are also contradictory experiences of being singled out, for example, among male pre-school teacher students, which means that they need to negotiate otherness, being in a minority position as a male student. The male nursing students seem to experience this to a lesser extent, but there is also the possibility of choosing part of the profession that supports a certain type of masculinity associated with risk and safety (ambulance nurse, emergency nurse), still connected to a certain kind of masculinity.

## Conclusions

This article stresses the importance of understanding that women and men in gender-segregated programmes experience their gender minority positions very differently. How gender is present differs significantly in different programmes. Using the concepts of “visibility,” “sense of belonging,” and “negotiating otherness” has made it possible to document how negotiation and belonging are part of students' everyday university lives, as is described by Sargent (2005) and others. The concepts were used to understand the effects of being repeatedly reminded of or being perceived as “different” in educational settings.

The following question was asked in this study:

How do women and men in gender-segregated, higher-education programmes experience their studies and study environment?

The study's most important contribution is its findings regarding the *differentiations in visibility*. A continuum of visibility experiences is explored, from men who receive positive attention to women who are being considered less knowledgeable and experience a study environment that is far from non-discriminatory and supportive. Our visibility scale indicates, as does previous research, that there are differences between how female and male students become visible, but *the differences can also appear within both groups of students*. This knowledge is crucial when designing interventions so as to provide positive study environments for both women and men (c.f. Kalev et al., 2006). The results suggest that universities should have different intervention strategies in their various programs (e.g., nursing, pre-school, construction engineering and computer science and engineering programmes) since the students' visibility experiences differ. The visibility scale could be used as a tool and starting point to identify expressions and differentiations in visibility experiences. In doing so, suitable interventions could be designed to provide non-discriminatory and supportive study environments for all students.

## Data Availability Statement

The datasets presented in this article are not readily available because consent was not obtained from the participants to share the transcripts of the interviews. Requests regarding the datasets should be directed to the corresponding author.

## Ethics Statement

Ethical review and approval was not required for the study on human participants in accordance with the local legislation and institutional requirements. The patients/participants provided their written informed consent to participate in this study.

## Author Contributions

All authors listed have made a substantial, direct and intellectual contribution to the work, and approved it for publication.

## Conflict of Interest

The authors declare that the research was conducted in the absence of any commercial or financial relationships that could be construed as a potential conflict of interest.
