# Supplementary material for: Differentiations in Visibility-Male Advantages and Female Disadvantages in Gender-Segregated Programmes
Source: Front Sociol. 2020 Oct 7;5:563204. doi: 10.3389/fsoc.2020.563204 (PMC8022530; doi:10.3389/fsoc.2020.563204)
Supplement: Supplementary file 1 [file Data_Sheet_1.PDF]

HÖGSKOLAN I HALMSTAD

Questionnaire: Differentiations invisibility

Introductory personal questions

Which program and which semester are you at?

Do you mind telling me how old you are?

Have you studied before or is this your first experience of university studies?

Introductory program-specific questions

Why did you choose to read this specific program?

Do you enjoy your studies?

What would you say is good about the program?

What do you think could be improved?

Program Content

How do you experience the content in the program?

Do you find the courses relevant for you and your future career?

What is your opinion about the different courses?

How do you view the relevance of / think about the structure of lectures and seminars?

How do you view the relevance of / what do you think about the course literature?

(Sometimes the interviewer made visible /asked follow-up questions on issues such as; are there any examples in the literature, at lectures and in examination assignments that you can relate to you and your experiences)

The gender division and its consequences

How do you experience the gender division in the program?

Are you affected by the gender division in your everyday life as a student?

(Follow up questions; are you pointed out - what is being normalized and what is perceived as deviant)

What experience do you have of belonging to a minority?

In what way has it affected you?

Do you have a strategy for dealing with situations that arise?

What do you think could be done to change the gender distribution in the program/ reduce dropouts?

hh.se

Högskolan i Halmstad • Box 823 • 301 18 Halmstad • Besöksadress: Kristian IV:s väg 3
Tel: 035-16 71 00 • registrator@hh.se • Org. nr. 202100-3203
